# Lessons from an intervention study on the sustainability of after-school comprehensive sexuality education in Zambia: the perspectives of teachers, health workers and guardians

**DOI:** 10.1186/s12978-024-01920-z

**Published:** 2024-12-18

**Authors:** Joar Svanemyr, Joseph Mumba Zulu, Ecloss Munsaka, Ingvild Fossgard Sandøy

**Affiliations:** 1 Independent Consultant, Oslo, Norway; 2 Centre for Intervention Science in Maternal and Child Health (CISMAC), University of Bergen, Bergen, Norway; 3 School of Public Health, University of Zambia, Lusaka, Zambia; 4 School of Education, University of Zambia, Lusaka, Zambia; 5 Centre for International Health (CIH), Department of Global Public Health and Primary Care, University of Bergen, Bergen, Norway

**Keywords:** Africa, Comprehensive sexuality education, Adolescent sexual and reproductive health, Youth, Sustainability

## Abstract

**Background:**

Comprehensive sexuality education (CSE) has been introduced in many sub-Saharan African countries, but limited political interest and insufficient funding have resulted in many CSE initiatives being dependent on donor funding or non-governmental organisations (NGOs) supporting its implementation. This has created concerns about the sustainability of the programmes. The objective of this study was to explore factors affecting the sustainability of CSE delivered through a youth club organized after school hours in Zambia.

**Methods:**

We interviewed teachers and community health workers (CHWs) who had implemented CSE as part of an after-school youth club set up as part of a cluster randomized controlled trial. The trial evaluated the effectiveness of economic support for adolescent girls, CSE and community dialogue meetings on adolescent childbearing. Teachers and CHWs in 63 schools were trained to facilitate the CSE youth clubs, and they were given economic incentives during the trial´s two-year intervention period to organize meetings every fortnight. Two years after the external support for the youth clubs ended, we conducted qualitative interviews with the facilitators in 15 of the 63 schools, interviews with some head teachers, and focus group discussions with guardians of adolescent girls.

**Results:**

Whereas CHWs were generally supportive of teaching adolescents about contraception, some of the teachers stressed that abstinence was the most effective method to avoid pregnancy and diseases. The respondents’ diverging points of view did not affect their willingness to continue teaching CSE, including contraception. However, the youth club meetings were only continued in a few schools after the external support period ended. This was attributed to transfers of trained teachers and a lack of training among the remaining staff; lapse of moral support, resources and incentives; limited involvement of the school management in the CSE initiative; and attention shifting to other projects.

**Conclusion:**

To ensure the sustainability of CSE initiatives for adolescents, emphasis should be placed on training several teachers in each school, and continued moral support and encouragement also appeared essential.

*Trial registration*: ISRCTN (ISRCTN12727868).

**Supplementary Information:**

The online version contains supplementary material available at 10.1186/s12978-024-01920-z.

## Plain Language Summary

This study looked at the sustainability of Comprehensive Sexuality Education (CSE) in Zambia, delivered through after-school youth clubs. These clubs were part of a larger project that also provided economic support to adolescent girls and organized community meetings.

The clubs were run by a teacher and a community health worker (CHW), who were trained and given financial incentives to hold meetings every two weeks. Two years after the project ended, interviews were conducted with various stakeholders.

While CHWs were generally in favor of teaching about contraception, some teachers emphasized abstinence as the best way to avoid pregnancy and diseases. Despite these differing views, all were willing to continue teaching CSE, including contraception. However, only a few schools continued the youth club meetings after the project ended. This was due to trained teachers being transferred, lack of training for remaining staff, loss of support and resources, limited school management involvement in the CSE initiative, and the need to focus on other projects. To ensure the sustainability of CSE initiatives, more teachers in each school should be trained, and continued support and encouragement should be provided.

**Supplementary Information:**

The online version contains supplementary material available at 10.1186/s12978-024-01920-z.

## Background

Comprehensive sexuality education (CSE), if well implemented [1], can equip learners with the awareness and skills they need to protect their health and develop good relationships in the transition to adulthood [2–5]. Over the past three decades, a number of initiatives to ensure that all children and youths receive CSE as part of basic education have been undertaken, and a review of sexuality education in 50 countries around the world indicated that 2/3 had made it compulsory [6]. The initiatives to introduce CSE in schools have been met with resistance from parents, politicians, religious leaders, community members and teachers in many African countries because of perceptions that discussing sexuality with children and adolescents is not compatible with local cultures and may encourage promiscuous sexual behaviour [7, 8]. A 2012 review of sexuality education curricula in southern and eastern Africa revealed substantial gaps [9], and in 2013, twenty countries in sub-Saharan Africa signed a declaration, committing to implementing education on comprehensive sexual and reproductive health and rights at the primary school level [10]. However, in practice, limited government commitment domestically, inadequate or non-existent budgeting, and weak monitoring and evaluation systems have affected implementation [11]. To fill these gaps, UN agencies and non-governmental organisations such as the International Planned Parenthood Federation (IPPF), Rutgers and Save the Children mobilized donor funding to strengthen CSE and supported curriculum development, training of teachers and provision of teaching materials in several countries [8, 12–14]. This has created concerns about the sustainability of CSE programmes when external support ends [15].

Zambia signed the 2013 Eastern and Southern Africa commitment to deliver comprehensive sexuality education and sexual and reproductive health services for young people to address the fact that Zambian adolescents face a range of SRH challenges, such as high rates of pregnancy, HIV transmission and child marriage [16–18]. Although most adolescent girls initiate sexual relationships before they are 17 years old [18], there is typically little communication between parents and their children about how to avoid pregnancy, and there are strong norms against adolescent contraceptive use [19]. As part of a general curriculum revision, the Ministry of General Education (MoGE) launched a comprehensive sexual and reproductive health curriculum for grades 5–12 in 2014. However, owing to resource limitations, a cascade model was chosen for the training of in-service teachers, where only a few were selected to be trained at the provincial level. They were then tasked with training teachers in their zones (subdivisions of districts), and the teachers trained at the zonal level were responsible for teaching the rest of the teachers in their schools. This affected the quality and intensity of the training, with teachers trained at the lower level receiving only a few hours of instructions on testable knowledge [20]. In addition, teacher manuals and other learning resources were not available during the first years of implementation, and when developed, they were not distributed in sufficient numbers to schools. These limitations contributed to studies conducted as late as 2017 reporting that many teachers still focused on encouraging sexual abstinence and left out information on contraceptive methods despite the new curriculum including this topic [21]. To address these gaps, NGOs such as Restless Development, FAWEZA, the Population Council, and the Planned Parenthood Association Zambia (PPAZ) have organized extracurricular CSE projects in selected schools [20].

This study builds on a cluster randomized trial on the effectiveness of CSE in combination with economic support and community dialogue on adolescent childbearing. The Research Initiative to Support the Empowerment of Girls (RISE) trial was conducted in rural basic schools in 12 districts in the Southern and Central provinces of Zambia. The formative research conducted prior to the trial, in 2014 and 2015, indicated that adolescent childbearing was common in the study districts [22]. Poverty, school dropout, community norms and misconceptions regarding sexuality and contraceptives were some of the reasons contributing to girls becoming pregnant early. Teachers in rural schools had not been trained in CSE and schools did not have any teaching resources to support them in delivering such education [23]. To achieve a link between the schools and the health sector while considering the low number of formal health workers in rural areas, one teacher and one Community Health Assistant (CHA) or Community Health Worker (CHW) were trained for each school to facilitate CSE-focused youth club meetings together. The interventions were implemented for 2 years beginning in September 2016, and during this period, the youth club meetings were held at the schools’ premises once every fortnight during the school terms. A snack and a drink were served at every meeting to motivate the learners to come to the after-school club, and the facilitators were given financial incentives for every youth club meeting. When the intervention period ended at the end of 2018, no further external support for the youth club was provided, and it was up to the schools to continue implementing such activities [23].

Process evaluation studies conducted halfway through the youth club implementation period indicated that teachers and CHWs perceived that the use of participatory approaches made learners active and increased their knowledge of CSE, assertiveness and self-esteem [24]. However, some teachers and CHWs experienced practical challenges with the youth club manuals they were instructed to use because certain concepts were difficult to understand and translate into local languages [25]. Cultural and religious beliefs among teachers and parents also complicated the delivery of reproductive health messages about contraceptives [24]. Interviews with learners who had participated in the RISE youth clubs, indicated that some misconceptions about contraceptives prevailed, and most of them did not view contraceptive use as appropriate for adolescents. According to the respondents, the youth club teachers emphasized abstinence and encouraged the learners to focus on school, and the interventions seemed to have reinforced rather than challenged community norms regarding appropriate behaviour for girls [26].

The primary objectives of the current sub study, conducted in 2020–2021, two years after the external support for the RISE youth club ended, were to explore (1) whether teachers had been motivated and able to continue providing CSE after the external support ended; and (2) factors leading to the continuation or disruption of youth clubs from the perspective of the involved teachers and community health workers. In addition, we explored guardians’ perspectives on youth clubs since the sustainability of CSE might partly depend on how guardians and other community stakeholders perceived it.

## Methods

### Design of the trial

The RISE trial was conducted in Mazabuka, Chikankata, Monze, Pemba, Choma, Kalomo, Chisamba, Chibombo, Kabwe, Kapiri Mposhi, Luano and Mkushi districts. A total of 157 rural basic schools were selected. Girls in grade 7 in 2016 were enrolled as participants, and they were followed up to the end of 2020. The age range of the participants at enrolment was 10–25 years, with a mean age of 14.1 years. The primary outcomes were early childbearing and the completion of basic education.

The 157 schools were randomly allocated to one of three arms: (1) *a control arm* (31 schools); (2) *an economic support arm* (63 schools) with cash transfers to girls and their families and payment of school fees in grades 8 and 9; and (3) *a combined intervention arm* (63 schools) receiving the same economic support combined with youth clubs focusing on CSE and community dialogue meetings.

Girls who participated in the trial and boys who attended the same class were invited to participate in the youth club, and they could continue in the youth club even if they quit school. The hypothesis linked to the youth club was that providing CSE could reduce sexual risk-taking, strengthen life skills and increase awareness of gender dynamics, which in turn could enable both girls and boys to make informed choices concerning sexual relations, choose appropriate protection methods, and thus reduce the risk of early childbearing. For each of the combined intervention schools, one teacher and one CHA or CHW were selected and trained as a team for 5 days prior to the implementation of the interventions in 2016. They also received a three-day refresher training after one year. The training provided to the facilitators focused on the CSE curriculum, value clarification, facilitation techniques and approaches to community mobilization. The aim of the training was to give them skills in using interactive teaching methods and to give them confidence in talking about sensitive topics. (Only a handful of the teachers had been trained in the official CSE/life skills curriculum through the cascade model by the MoGE prior to the RISE training). The facilitators were also provided with a detailed manual with instructions for each of the 36 youth club sessions on what information to provide, what activities to include, and what the take home messages should be for every session. The following topics were covered in the manuals: the benefits of education; school re-entry; the risks of early pregnancy, early marriage; puberty and menstruation; gender roles; healthy relationships and communication; peer pressure; sexual desires; self-esteem and assertiveness; decision making and setting goals; and ways to prevent pregnancy, HIV and other sexually transmitted infections. The manual was designed by the RISE team and was based on the Tuku Pamoja Adolescent Reproductive Health and Life Skills Curriculum, produced by PATH and the Population Council [27]. Abortion and homosexuality were not included, as they were regarded as too sensitive topics in the Zambian context. The most common learning activities were interactive discussions between the facilitators and the learners, group discussions, and role plays. In addition, two fiction films were produced by the project. One film focused on the value of education, whereas the other focused on the risks of early pregnancy and early marriage.

Although the manual was in English, the facilitators were instructed to hold the youth club sessions in a local language to accommodate pupils not fluent in English. The teachers were given ZMW 125 (USD 12.5 at the time) and the CHA/CHW ZMW 100 (USD 10) for every youth club meeting held to ensure that all the sessions were implemented. The schedule for the meetings was prepared by the project’s Trial Supervisors so that they could monitor at least 20% of the meetings at each of the schools. During the monitoring visits, the Trial Supervisors used a checklist to record whether the manual was followed and how well the facilitators delivered the content and organized the activities. Feedback on performance was given to the facilitators after every monitoring visit.

The teachers and CHAs/CHWs were also engaged in facilitating community meetings for guardians and other community members. Community meetings were held twice per term in each school and promoted supportive social norms around the postponement of early marriage and early childbearing, as well as education for girls.

### Study design

This qualitative study was based on in-depth interviews (IDIs) with youth club facilitators and head teachers and focus group discussions (FGDs) and interviews with guardians of adolescents who had participated in the RISE youth club. It was conducted between September 2020 and March 2021, i.e. 2–2.5 years after the youth club implementation support ended.

### Study setting

The study was conducted in 24 of the 63 schools that were part of the combined intervention arm. We selected some schools that had good attendance of the youth club and some where the attendance was low, and some that were remote and some that were closer to the district centres.

### Sampling

We planned to invite the teachers and CHAs/CHWs who had facilitated the youth club meetings in 15 of the 63 combined intervention schools to take part in the interviews. To explore guardians’ perspectives, focus group discussions (FGDs) were conducted in four of the selected schools with 4–5 guardians in each, and in the other eleven schools we interviewed 2 guardians per school, aiming to get a mixture of guardians of girls and boys who attended the youth club, including some who attended most of the community meetings and some that rarely came to the community meetings. We had also planned to interview head teachers in 1/3 of the 15 schools. However, in several of the selected schools the head teacher had recently been employed (and had limited knowledge of RISE) and the previous head teacher had been transferred to another school and had little knowledge about what happened afterwards. Thus, we purposively sampled another nine head teachers who had been at other combined intervention schools since the trial started and were expected to be knowledgeable about the RISE youth club.

### Data collection

The interviews were conducted by the third, second and first authors and two research assistants, all experienced in qualitative research methods. The FGDs were moderated by the third author and one of the research assistants. Both the interviews and the discussions were guided by semi structured interview guides. Teachers, guardians, and CHWs were interviewed face-to-face, whereas head teachers were interviewed by phone or face-to-face. RISE teachers and CHAs/CHWs were asked to share their experiences from teaching CSE in the youth club, whether they had continued teaching CSE and what would be required to continue with the youth club. Head teachers were asked how sexual and reproductive health education had been organised in the school after RISE ended, what contributed to the youth club not being sustained, and what support schools need to implement CSE. In the interviews and discussions with parents the questions focused on what their child had told them about the youth club, their opinion on teaching adolescents how to avoid pregnancy, and their views on discussing sexual and reproductive health issues with their children. The interviews with teachers and CHWs were conducted in English since all of them were conversant in the English language. The interviews with guardians were conducted in the local language of their choice. All respondents were assured confidentiality. The names of the respondents were not recorded, and they were informed that we would not share any of information in any publications that could identify them, such as the name of the school they were linked to.

### Data analysis

The interviews in English were transcribed verbatim, and the interviews in local languages were translated and transcribed by people who were fluent in the local languages. To ensure content validity, the first author verified the transcripts of the interviews conducted in English by listening to the audio and comparing them with the transcripts. The transcripts were imported into NVivo software version 12 for qualitative analysis and coded by the first author with a mix of preestablished and emerging codes—representing meaning units. The codebook was approved by all the authors. Thematic analysis was employed with codes being merged into themes, and these were synthesized into written text and reviewed by all co-authors.

## Results

We ended up with interviewing 14 teachers and 12 CHAs/CHWs from the 15 initially selected schools as some of the facilitators were not available at the time of the study. In addition, 15 head teachers and 22 individual guardians (parents, grandparents and sisters of children who participated in RISE) were interviewed and four FGDs with guardians were conducted. The sociodemographic characteristics of the respondents are presented in Supplementary Table 1.

The findings are presented according to the research objectives.

### Teachers’ and CHWs’ experiences with training and facilitating youth clubs

All the teachers and CHWs expressed satisfaction with the training they received, and they felt well prepared to apply interactive methods and to teach the content of the CSE sessions. They were also very positive about their experiences from the youth clubs and indicated that they wished they could have continued and included more adolescents. All of them stated that the CSE had been a positive experience and that the learners enjoyed the meetings and the role plays, and that they felt that the learners had become more assertive. Teachers reported that the use of interactive methods such as role plays and group discussions helped the youths comprehend and remember the messages, and several informants emphasized that role plays helped shy and reserved learners to be more active and outspoken. The two films made by RISE were also remembered as much appreciated, and the adolescents had understood the messages about early pregnancies being associated with risks of health complications and unhappy marriage, and about the benefits of completing school.

The facilitators were divided concerning the inclusion of contraception in the youth club manual. Some teachers found it challenging to talk about contraceptives, either because of lack of knowledge or because they feared it would encourage the pupils to become sexually active. Other teachers felt that learning about contraception is okay for those who are not able to abstain, whereas most community health workers believed that all adolescents should be taught about contraception and provided access.

The great majority of the teachers and CHWs said they would like to continue teaching CSE and life skills. However, beyond covering the relatively limited SRH content integrated in various subjects, in accordance with the Ministry of Education's (MoE) curriculum, only two of the interviewed head teachers said they had taken initiatives to continue providing CSE as an after-school activity for learners after the RISE support ended. The motivating factor was the perception that the learners had benefitted from the CSE by becoming more focused on school work and engaging less in romantic relationships.

One of the head teachers, who claimed to have continued with a youth club, explained:

R: We are continuing with the youth clubs and also have it included in the curriculum. We have so many subjects, so we shifted them to the youth club we have every Wednesday.

I: Have you continued using the RISE manual?

R: We have books from the resource centre, life-skill books. Some topics from the RISE manual are so good so we combine them. *(Head teacher, site no. 12)*

A few schools had established new after-school youth clubs initiated by other organisations focusing on CSE or HIV prevention and expressed that the content of these overlapped with the RISE youth club curriculum:

Another programme that was introduced in the school that was being guided by DREAMS, in particular their area of expertise, was comprehensive sexuality education, and even up to today, that programme is still there in the school. *(Teacher, site no. 9)*

... DAPP, the one for clothes, they have a club where they teach about HIV/AIDS, and the importance of education. *(Teacher, site no. 14)*

### Guardians’ perspectives on youth club content

Guardians were overall positive towards the youth club because they thought their children had learned about the importance of education, focused more on school, and had stayed away from sexual relationships that could have led to unplanned pregnancy and school dropout. Some guardians were positive about the youth club curriculum covering contraception, and they supported adolescents’ contraceptive use because they believed that adolescents cannot be expected to always abstain from sex; thus, they need protection.

I feel better when they are taught. I say so because they do some things out of ignorance, but when a person is taught and they know what’s right and wrong, they will be able to make decisions based on what they feel is best for them. (*Male parent, site no. 12)*

However, other guardians were more critical about facilitators talking about contraceptives and believed it would turn the girls into ‘prostitutes’, and most of the guardians emphasized that abstinence was best. Many teachers and CHWs also talked about parents who reacted negatively to the sessions about contraception.

The one on condoms received negative comments, because parents were saying that we were teaching pupils on prostitution. *(CHW, site no. 11)*

A few teachers had experienced parents asking them to stop talking about contraception, but they had been successful in convincing these parents that CSE would be beneficial to the adolescents and help them protect themselves against pregnancy. Other parents had reportedly changed their opinions about the CSE provided in the youth clubs after the community meetings, and some had even become supportive of contraceptive use:

Because of that topic which parents learnt on the importance of education, they were more supportive of their children’s education. They saw the beauty of education and so in order for their children to complete their education, parents said, “Let them go”. Some of the parents even encouraged their children to go and get the injection, while others even take their daughters themselves. You find that a mother has brought her daughter to the clinic for the injection. *(CHW, site no. 17)*

### Factors leading to the discontinuation of youth clubs

Despite all the positive experiences and benefits reported, out of the 24 schools from which the informants in this study came, only three schools had decided to continue with the youth club in some form. Some schools had ‘HIV/AIDS clubs’ with much less comprehensive content. In the rest of the schools, sexuality education after the RISE club ended was only reported to be delivered in accordance with the official school curriculum, integrated into different subjects. The reasons for discontinuation according to the teachers included a lack of economic and material resources, trained teachers being given other responsibilities or being transferred to other schools, a lack of trained staff among those remaining at the school, and a lack of training and involvement of head teachers.

Even though the head teachers’ involvement and engagement were limited during the implementation of the RISE youth club, all the teachers said that the head teachers had been supportive and provided them with room and materials to run the RISE youth club. A few of the head teachers had attended youth club meetings and actively encouraged the pupils to participate. Nevertheless, none of the teachers or community health workers reported that the head teacher had taken initiatives to facilitate continuation of the youth club meetings after the RISE support ended. Some teachers claimed that they would have been willing to continue with the youth club if the head teacher had put it in the programme of the school.

Some head teachers, teachers and CHWs emphasized the lack of resources. The schools were not provided with and could not afford to procure relevant teaching materials such as manuals and books.

I: Any additional support you may need?

R: Maybe just material support. The literature, we do not have many to cater for everyone. You may find that you have only 3 to 4 books when teaching and the pupils are many.” *(Teacher, site no. 9)*

Some were of the opinion that continuing the youth club with sexuality education would require funds to continue providing snacks and economic assistance to learners since this habit was introduced by RISE:

At the end of the meeting, they were given some snacks. Now, when RISE stopped the sponsorship, it’s like now they were not motivated. That’s after sharing, after the lessons, we are like ‘we’ll meet next time”. Now, there were no snacks. So like that—it’s like learners could not be motivated further. *(Head teacher, site no. 9)*

It is the issue of the precedence because when they were gathering, they used to have snacks and so forth. You know when you set a precedence and then you do not have resources, people will start shunning away.* (Head teacher, site no. 8).*

The attention of the schools also moved to other projects with funding, and several head teachers did not even consider continuing the RISE youth club. In their opinion it was not possible without continued training and encouragement.

Like I said, usually people when they are told that this thing will end at such a point, then they think that phase of that programme has ended, just as other programmes that came in education. It comes for 5 years, and then it ends; it’s forgotten. So, because there are many projects that have been coming in education. Some of them they are piloting. Some of them they are just programmes supported by certain NGOs, and when those NGOs go, you find that that project ends. *(Head teacher, site no. 24)*

When asked how initiatives that are started by external actors can be made sustainable, one head teacher said:

How we can do that? First, to continue training people, continue reminding us. Because you know when the initiator leaves, then the people tend to relax. So, if we can continue interacting like this, you give us programmes, come up with some more programmes, sending words of encouragement to the members and to the pupils, it would be better than just leaving us here. It’s not easy because there are so many programmes and so many organizations. *(Head teacher, site no. 10)*

Some noted that this was linked to expectations created by RISE that facilitating the youth club would be remunerated:

I think it still comes back to the issue of resources. Because when this youth club was going on, remember the person who was in charge was a teacher, and [he] was getting something. Because as the head of the school, who was on the observing side, who could see what was happening, it is like the person who was in charge of the meeting was very much interested in conducting those meetings because, at the end of the day, something was coming in his pocket. So, immediately that funding got stopped, then everything died just like that. *(Head teacher, site no. 22)*

Some teachers said that they did not continue teaching sexuality education because they were given other responsibilities, including teaching lower grades. Some head teachers mentioned that the trained teacher had been transferred to another school, and since RISE trained and incentivized only one teacher at each school, there was no one to continue teaching when that person left or received other responsibilities. Training of more teachers would be required to make it more sustainable.

You know, out of the 32 teachers I have, only one attended the training. So even when that person comes to give feedback to the rest, we don’t expect people to be well informed at once. So if we can have a very good number that can be trained, then the better. They can understand it more. *(Head teacher, site no. 10)*

The head teachers noted that they did not receive training and were not involved in the coordination or monitoring of the meetings. Instead, this was done by the project staff.

I think the head teacher should also be trained so that in case the teacher gets transferred, the head teacher should remain with the knowledge on how to run the project. (…) When the teacher got transferred, the head teacher could have had knowledge to start another one and get another teacher and give that teacher the knowledge on how to run the project. I think that would have helped. *(Head teacher, site no. 23)*

The fact that the head teachers were not actively involved made them feel that they were not supposed to contribute to the club or its sustainability. They also expressed that they would appreciate to be trained by experts, not by their own teachers.

The simple answer is that do not leave him [the head teacher] [out] of important things. If you don’t involve him, others would understand, but others would say “Ah, after all they don’t call me for this. They want me to coordinate, to supervise, but they don’t call me.” Try to involve them on the most important things involved in the programme. For example, availing the materials that are there and up to date with what is going on and involving them when there is need for capacity building as opposed to second-hand information. Avoid second-hand information for the head teacher. I would prefer that he is there. It’s better. *(Head teacher, site no. 8)*

This same head teacher stated that the deputy should also be included:

You know most of the programmes in Africa and Zambia have been leaving out the office of the deputy head. The deputy head is the engine of the school and should be there. And once you side-line that, then there is a problem. The head teacher is sometimes busy, and so in the process, we lose grip on the programme. Thus, the deputy head teacher should be involved. *(Head teacher, site no. 8)*

Indeed, a common recommendation for future projects was to involve the school management to keep teachers motivated to provide CSE:

I think that one starts with us supervisors too. If I am very active in this area as a supervisor, thanking those who are doing very well and during assemblies, we find out what they know class by class, I think teachers—though not mandatory—will be forced to work hard and compete within the school. This may bring change. So teachers are not very difficult to motivate. It is just a matter of making a programme and supervise with the head teacher in the forefront. *(Head teacher, site no. 18)*

## Discussion

Two years after the support for implementing CSE as part of the RISE youth club ended, the facilitators expressed high levels of satisfaction related to the experience. They were very positive about the content and teaching process and stated they had observed clear benefits for the learners from the youth clubs. All the teachers and CHWs said they would have liked to continue teaching CSE and life skills. The guardians would also have liked the CSE to continue despite some of them articulating resistance and scepticism in the early phases, particularly concerning teaching about contraception. The interviewed informants reported a variety of reasons why the youth clubs had not continued at their schools. The teachers mentioned that they had other responsibilities, and that the school management did not provide support or express that continuation should be prioritized. Some believed that continuing with the youth club would have required incentives to motivate the learners who had become accustomed to obtaining snacks and drinks. The fact that incentives were no longer provided to the facilitators was also regarded as a barrier. In addition, the head teachers pointed out that RISE trained only one teacher at each school, leaving the school vulnerable to transfers of teachers and changing responsibilities because the other teachers did not have the competence required to take up this task. In addition, since the project did not involve head teachers in the training or monitoring of the youth club, they did not feel that they had any responsibility to bring the youth clubs forward.

Guardians’ perspectives of the youth club varied, but they all expressed positive views regarding the content on the benefits of education. Contraceptives was a more contentious topic, and parental concerns about this was reported also in interviews we conducted during the implementation period [24]. However, the fact that several of the interviewed guardians expressed appreciation that the adolescents learned how to protect themselves if they were sexually active is in line with previous studies that indicate that guardians may be convinced that CSE is beneficial when sufficient information on what is taught is provided [28]. Nevertheless, the views of guardians did not appear to have influenced the schools’ decision (not) to continue the youth club, and may reflect that there had been little discussion about CSE between guardians and teachers after the RISE youth club implementation ended as other issues were probably regarded as more important.

Introducing various approaches to prevent unintended pregnancy is an essential part of CSE, but there could be a risk that scepticism among facilitators regarding the inclusion of modern contraception might negatively affect the sustainability of CSE. The teachers and CHWs interviewed for this study expressed somewhat different views on teaching adolescents about contraception. Several teachers who admitted that they told the adolescents that they should abstain from sex until they finish school, claimed that they also taught them about contraception. This corresponds with findings from South Africa, where teachers attempted to deliver a ‘hybridized perspective’ through a strategical combination of promoting abstinence with teaching about contraception and safe sex when delivering mandatory Life Orientation (LO) education [29]. Allowing teachers to use their ‘discretion’ to adapt the curriculum to personal and local concerns in such ways might increase sustainability but may conflict with the intention of CSE and carry the risk that the effect of the programme may decrease [12, 21].

The positive views expressed by the teachers on interactive teaching stand in contrast to findings from other studies from various contexts in Asia and Africa that have found that teachers often limit skills-building activities because they believe that pupils will find them difficult or that they themselves do not have sufficient confidence to facilitate them [12, 30]. The RISE teachers indicated that they had previously learned about interactive teaching in their teacher training, but evaluations of the implementation of the official CSE curriculum indicate that lack of training and dedicated time to teach CSE, result in many Zambian teachers still sticking to traditional lecturing as the dominant teaching mode for life skills and sexuality education with limited possibilities for pupils to ask questions [31]. The fact that the two RISE trainings the teachers and CHWs attended focused on facilitation skills in delivery of CSE is likely to have boosted their confidence to be more interactive [25]. The importance of proper training to gain confidence to teach sensitive sexuality topics has also been documented in other studies [32, 33]. In addition, access to CSE teaching materials has been found to be a challenge for the implementation of the official Zambian life skills/CSE curriculum [31], and the detailed instructions regarding activities in the RISE manual also appeared to make it easier for the facilitators to cover sensitive topics in an age-appropriate manner.

The lack of resources and programmatic support after the external support ended contributed to both the teachers and the school management teams turning their attention to other tasks and opportunities. This finding suggests that incentives are helpful to achieve rapid implementation but may prevent the institutionalization of a programme. For the sake of sustainability, it is likely that it would have been a better investment to train several teachers instead of providing incentives. The lack of continuity adds to concerns about the sustainability of such programmes when they are initiated, organized and financed by external actors. Schools in sub-Saharan Africa often have a “patchwork of NGO initiatives”, which means that once a project has ended, teachers and school management move on to spend their time and attention on the next one [15].

The reported experiences from this study are likely to be relevant for initiatives by non-governmental organizations and others that introduce CSE programmes with limited funding periods, creating a need to use mechanisms that can ensure rapid implementation and the need to set up a monitoring system outside the MoE structures to document outputs and outcomes that are achieved. Regular monitoring is usually needed to ensure sufficient implementation quality [11, 34]. However, our findings indicate that more emphasis on building functioning monitoring mechanisms within the routine system may be preferable to ensure both continuation and quality if long-term sustainability is desirable. CSE initiatives from the government are better placed to be sustainable because they can incorporate monitoring of CSE in the ordinary supervisory mechanisms of schools. It should be noted that existing supervision in the Zambian school system is relatively weak due to limited funding [31]. The advantage of the CSE model that the MoE in Zambia aims to implement is that it is integrated into various subjects—as recommended by UNESCO—such as integrated science, biology, civic education, religious education, home economics, and social studies—and aims to train all Zambian teachers in CSE. This model is likely to be more sustainable but does require more substantial investments in good-quality training and teaching resources than the Zambian government invested in the first years [20].

This evaluation was conducted in a large proportion of the 63 schools that had implemented the RISE youth club, and we made a deliberate effort to include a great variety of schools. Thus, we believe that the findings are transferable to other schools that implemented the RISE youth clubs. Since the interviews were conducted approximately two years after the intervention ended, there might have been some recall bias resulting in underreporting of negative aspects. Second, due to the COVID-19 pandemic, it was not possible to visit all the research sites and conduct face‒to-face interviews, which meant that some of the informants had to be interviewed via phone. While being efficient in terms of the time used by the informant and the interviewer, phone interviews have the limitation of minimizing the personal interaction between the two. This lack of personal touch may have reduced openness on the part of some of the informants. Third, the high rates of transfers among teachers in Zambia also affected the teachers involved in the implementation of the RISE project. This meant that we had to limit our collection of data to those schools where the original RISE teachers and head teachers were still present, except for two cases where we were able to contact and interview head teachers who had moved to another school. It is likely that teachers were extra positive toward the youth club because they appreciated the close monitoring and remuneration provided by the project since such support was not provided to teachers implementing the national life skills/CSE curriculum, and the guardians may have made positive statements about the youth club because they appreciated the economic support and the community meetings. Fortunately, the informants appeared to be frank about why the youth clubs had stopped, sharing multiple reasons and pointing out weaknesses in the implementation strategy. The consistency of the responses from health workers, teachers and head teachers most likely indicates that the findings are trustworthy.

## Conclusion

The youth club offering after-school CSE was regarded as beneficial to adolescents both by the facilitators and guardians. Despite teachers being motivated to continue with CSE, there were several barriers to the sustainability of the externally funded initiative. To ensure long-term continuation of CSE to adolescents, emphasis should be placed on high-quality training of a substantial proportion of teachers and adequate material resources and on continued supportive supervision, including from the school management. The use of economic incentives to motivate facilitators and learners may be counterproductive in the long run.

## Supplementary Information

Additional file 1.

## Data Availability

The data analysed in the current study are available from the corresponding author upon reasonable request.

## Abbreviations

CHW: Community health worker
CSE: Comprehensive sexuality education
MoGE: Ministry of General Education
MoE: Ministry of Education
NGO: Non-governmental Organization
SRH: Sexual and reproductive health
RISE: Research initiative to support the empowerment of girls
