# Supplementary material for: Lessons from an intervention study on the sustainability of after-school comprehensive sexuality education in Zambia: the perspectives of teachers, health workers and guardians
Source: Reprod Health. 2024 Dec 18;21:191. doi: 10.1186/s12978-024-01920-z (PMC11656948; doi:10.1186/s12978-024-01920-z)
Supplement: Supplementary file 1 — Additional file 1. [file 12978_2024_1920_MOESM1_ESM.docx]

**Supplementary table 1: Sociodemographic profile of respondents**

| **Data collection method** | **Respondent category** | **Number of schools** | **Number of respondents** | **Mean age (range) in years** | **Sex** | **Educational level range** |
| --- | --- | --- | --- | --- | --- | --- |
| IDI | RISE teachers | 14 | 14 | 41 (30-52) | 4 males, 10 females | Diploma - master's degree |
| IDI | CHAs/CHWs | 12 | 12 | 48 (27-57) | 4 males, 8 females | Completed grade 7 - grade 12 |
| IDI | Head teachers | 15 | 15 | 53 (50-57) | 12 males, 3 females | Diploma - master's degree |
| IDI | Guardians | 11 | 22 (2 grandparents, 2 sisters, 6 fathers, 12 mothers) | 49 (24-65) | 7 males, 15 females | No education - Diploma |
| FGD | Guardians | 4 | 19 | 44 (27-59) | 5 males, 14 females | Not recorded |
